# Wound of External Carotid Artery

**Published:** 1854-06

**Authors:** J. W. B. Reynolds

**Affiliations:** New Market, Franklin county, Missouri


### ECLECTIC DEPARTMENT.

### *Wound of the External Carotid Artery, cured by Compression and Styptics.* By J. W. B. Reynolds, M. D., *of New Market, Franklin county, Missouri.*

I have recently had a case under my care, which, though it may present nothing new, or extraordinary, is, nevertheless of rare occurrence, and altogether, of sufficicient importance to merit a public notice—illustrating as it does, the advantage of perseverance under the most trying and discouraging circumstances.

A slave belonging to Mr. A. R., a wealthy planter of this county, was assaulted by a couple of other negroes—the one holding him whilst the other stabbed him several times in the back, and ended by cutting his throat. The hemorrhage was profuse, and he was placed under the temporary charge of a couple of young physicians who were on the ground. A messenger was immediately dispatched for me, about nine miles. I reached the patient as soon as could be expected, considering I had a dark night and bad road, and found him weak, cold, and almost exhausted from loss of blood. Every garment upon him was completely saturated. The wound in the throat was not bleeding at the time of my arrival, the hemorrhage having been arrested by the physicians in charge, by compression, &c. They informed me, that it was altogether venous. I closed the large gaping wounds in the back by sutures, and let the patient remain quiet until morning, when he was conveyed on a bed placed in a carriage, to his master's house. For three or four days he did very well, and appeared in a fair way to recover—regaining his strength sufficiently to walk about the room and yard.

Just when I imagined that all danger was passed, I was summoned in great haste in the night to visit the patient again, the messenger informing me that profuse and alarming hemorrhage had recurred. On my arrival, I found a gentleman belonging to the family, compressing the wound with his fingers—thus keeping the hemorrhage under temporary control. On removing the pressure, the blood jetted out with a hissing noise which could be heard by all the bystanders. The blood was ejected per saltum and with great force, which at once convinced me that it proceeded from an artery, and that one of considerable magnitude. The wound being deep behind the angle of the jaw, I thought at first it might proceed from the facial branch of the external carotid, but upon a more careful examination I was satisfied it came from the caroted itself. Here was a bad, an almost hopeless case. The patient was again cold, almost pulseless, and weltering in blood—fresh from the main reservoir of life. There was little time to lose; it was almost impossible to tie the artery at the seat of injury, and there was still a hope that compression and cold might succeed.

These I employed; closing the wound, applying a graduated compress, and keeping it constantly wet with cold water. For twenty four hours the patient did well—the hemorrhage appearing under complete control. At the end of this time it again recurred. I removed the dressing, applied lint wet with a styptic solution, and dressed as before. In twelve hours there was another attack of bleeding. I now filled the wound with pledgits of lint, wet with a saturated solution of alum—applied the compresses and kept them wet with a cold infusion of nut galls, and a solution of tannin. The hemorrhage still recurred, in spite of all efforts, about every twelve hours, and though not so profusely, I knew my patient would not be able to support this drain much longer. I now determined as a dernier resort to ligate the common carotid. This being a formidable operation, I had deferred it until the last moment, and determined not to resort to it, until I had exhausted every other means, and the condition of the patient absolutely required it. Accordingly, I once more applied the dressings, and left a careful assistant to stand by the patient, and keep the dressings constantly wet with the cold astringents before mentioned; while I left to make the necessary arrangements for the operation.—On my return next morning for this purpose, I was gratified to find that the hemorrhage had not returned, and on examining the wound, I found it filled with a firmer and better clot than any I had yet seen. Whatever eclat might have been gained by the operation, thinking it much better surgery to cure the case without it, if possible, I determined to persevere with the means I had been using. On my visit next day there had still been no recurrence of hemorrhage. Next day the same, and the day following the wound was suppurating. From this time every thing went on well; the wound rapidly filled with granulations, and the man is now well.

To account for the arterial hemorrhage in this case, several days after the infliction of the wouud, I supposed that the external coat of the artery was divided by the knife, leaving the internal ones entire, and when reaction was perfectly established and the vessels had again becôme distended with blood, these coats gave way. Whether I was justified in relying for nearly two weeks (with hemorrhage every day) on the remedies which finally succeeded, and neglectig an operation, is a debateable question. I am at least satisfied with the result, and would treat another case in the same way.

As for the styptics I used, cold water might have answered the same purpose. Using them cold, I knew in this case, they would be as good as water alone, and I thought they might be a little better.—*St. Louis Med. & Surg. Journal.*

